# QueF-Like, a Non-Homologous Archaeosine Synthase from the Crenarchaeota

**DOI:** 10.3390/biom7020036

**Published:** 2017-04-06

**Authors:** Adriana Bon Ramos, Lide Bao, Ben Turner, Valérie de Crécy-Lagard, Dirk Iwata-Reuyl

**Affiliations:** 1 Department of Chemistry, Portland State University, Portland, OR 97207, USA; efectegerundi@gmail.com (A.B.R.); bold@imau.edu.cn (L.B.); beturner@pdx.edu (B.T.); 2 The Department of Microbiology and Cell Science Department, University of Florida, Gainesville, FL 32611, USA; vcrecy@ufl.edu

**Keywords:** archaeosine, biosynthesis, modified nucleoside, tunneling-fold superfamily, amidinotransferase

## Abstract

Archaeosine (G^+^) is a structurally complex modified nucleoside ubiquitous to the Archaea, where it is found in the D-loop of virtually all archaeal transfer RNA (tRNA). Its unique structure, which includes a formamidine group that carries a formal positive charge, and location in the tRNA, led to the proposal that it serves a key role in stabilizing tRNA structure. Although G^+^ is limited to the Archaea, it is structurally related to the bacterial modified nucleoside queuosine, and the two share homologous enzymes for the early steps of their biosynthesis. In the Euryarchaeota, the last step of the archaeosine biosynthetic pathway involves the amidation of a nitrile group on an archaeosine precursor to give formamidine, a reaction catalyzed by the enzyme Archaeosine Synthase (ArcS). Most Crenarchaeota lack ArcS, but possess two proteins that inversely distribute with ArcS and each other, and are implicated in G^+^ biosynthesis. Here, we describe biochemical studies of one of these, the protein QueF-like (QueF-L) from *Pyrobaculum calidifontis,* that demonstrate the catalytic activity of QueF-L, establish where in the pathway QueF-L acts, and identify the source of ammonia in the reaction.

## 1. Introduction

**Figure 1:**
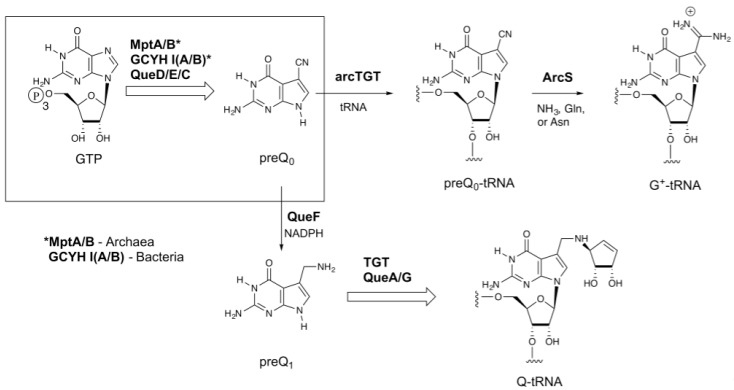
The biosynthetic pathways to 7-deazaquanosine modified nucleosides of transfer RNA (tRNA). The upper branch occurs in the Archaea and leads to archaeosine (G^+^), while the lower branch occurs in bacteria and leads to queuosine (Q); the boxed region is common to both. *MptA/B catalyze the initial steps in Archaea [21,22], while GCYH IA or IB catalyze the initial step in bacteria [23,24]. ArcS: Archaeosine Synthase; preQ_0_: 7-cyano-7-deazaguanine; preQ_1_: 7-aminomethyl-7-deazaguanine.

Transfer RNA (tRNA) is unique among nucleic acids in undergoing extensive nucleoside modification during maturation of the transcript. Over 100 modified nucleosides have been characterized [1], and these typically comprise roughly 10% of the nucleosides in a given tRNA, but can account for as much as 25% [2]. The 7-deazaguanosine nucleosides archaeosine (G^+^) and queuosine (Q) are two of the most structurally complex modified nucleosides found in tRNA [3]. Both share a 7-deazaguanosine core (Figure 1), but differ in the extent of further elaboration; archaeosine possesses an amidine functional group at the 7-position [4] of this core structure, while queuosine possesses a cyclopentenediol ring appended to an aminomethyl group at the 7-position [5,6], which in some mammalian tRNAs can be glycosylated with galactose or mannose at one of the hydroxyls of the cyclopentenediol ring [7,8], or in some bacteria aminoacylated with glutamate [9,10,11,12]. Queuosine is ubiquitous throughout eukaryotic and bacterial phyla and occurs exclusively at position 34 (the wobble position) in the anticodons of tRNAs coding for the amino acids asparagine, aspartic acid, histidine, and tyrosine [13]. Its location in the anticodon suggests a role in modulating translational fidelity and/or efficiency, and physiological studies are consistent with such a role [14,15,16,17]. In marked contrast, archaeosine is present only in archaeal tRNA, and is located at position 15 (and position 13 in *Thermoplasma acidophilum* tRNA^Leu^ [18]) in the dihydrouridine loop (D-loop) [19], where it is proposed to play a key role in tertiary structure stabilization [20].

Archaeosine is found quasi-universally along the archaeal tree, and while all Archaea that possess G^+^ possess genes encoding homologs of the biosynthetic enzymes up through arcTGT, the enzyme that catalyzes the exchange of the genetically encoded guanine for the precursor 7-cyano-7-deazaguanine (preQ_0_) in tRNA (Figure 1), Archaeosine Synthase (ArcS), which is the amidinotransferase responsible for converting preQ_0_-tRNA to G^+^-tRNA [25], is not universally distributed. *arcS* is ubiquitous in Euryarchaeota, but the majority of sequenced Crenarchaeota lack *arcS* homologs. Previously we identified two non-homologous enzymes that, when expressed in *Escherichia coli,* resulted in the production of G^+^-modified tRNA [26]. One of these, GAT-QueC, is a fusion of a glutamine amidotransferase domain (GAT) with QueC, the enzyme responsible for forming the precursor preQ_0_. Interestingly, the other enzyme, QueF-like (QueF-L), is a homolog of the bacterial enzyme QueF, which catalyzes the NADPH-dependent reduction of preQ_0_ to 7-aminomethyl-7-deazaguanine (preQ_1_) [27] in the queuosine pathway (Figure 1). QueF is a member of the tunneling-fold (T-fold) superfamily [27,28], a small structural superfamily that was already known to support an astonishing diversity of chemical reactions [29].

Recently, we reported the X-ray crystal structure of QueF-L [30], confirming that it is a member of the T-fold superfamily with significant structural homology to QueF. Here we describe biochemical studies of the recombinant QueF-L enzyme from *Pyrobaculum calidifontis* that clearly demonstrate the catalytic activity of QueF-L, establish where in the pathway QueF-L acts, and identify the source of ammonia in the reaction.

## 2. Results

### 2.1. Over-Expression of *queF-L* and Purification of Recombinant *Pyrobaculum calidifontis* QueF-L

The *queF-L* gene from *P. calidifontis* was synthesized (GenScript) with codon optimization for *E. coli* expression. We subcloned *queF-L* from this construct into a pET30-based vector for over-production of QueF-L as a His_6_-affinity tagged recombinant protein. The protein was expressed well, and was purified to >95% homogeneity by an initial heat treatment (80 °C) to precipitate heat labile proteins followed by affinity chromatography on Ni^2+^-nitrilotriacetic acid (NTA) resin.

### 2.2. Identification of the Substrates for *P. calidifontis* QueF-L

**Figure 2:**
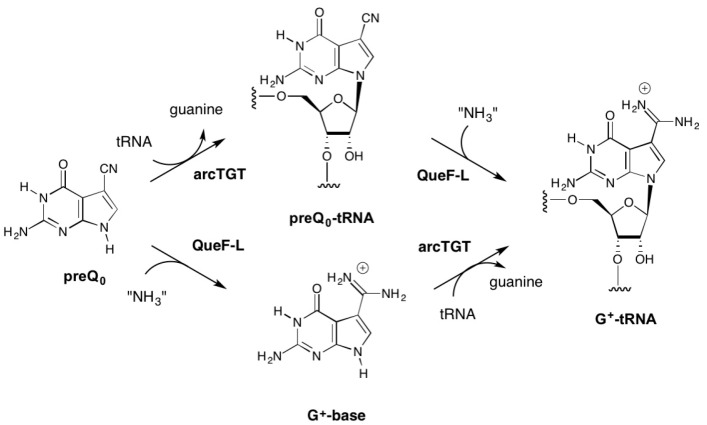
Possible routes to G^+^-tRNA in Crenarchaeota possessing QueF-like (QueF-L) enzymes. The top pathway is analogous to the known pathway in Euryarchaeota that utilizes ArcS to carry out the amidation of preQ_0_-modified tRNA. The bottom pathway is analogous to the known reaction of bacterial QueF, which carries out the four-electron reduction of the nitrile group in preQ_0_ to give the aminomethyl product preQ_1_.

While our in vivo data clearly demonstrated that QueF-L functions as an amidinotransferase in the biosynthesis of G^+^-modified tRNA, even when expressed in *E. coli* [26], it was not known if the conversion of the nitrile to the formamidino group occurred before or after preQ_0_ is inserted into tRNA (Figure 2), or what the source of NH_3_ was.

Given that ArcS functions on preQ_0_-modifed tRNA in the final step of G^+^ biosynthesis (Figure 1), it was reasonable to propose that QueF-L functioned analogously (Figure 2, top). On the other hand, QueF acts on free preQ_0_ in bacterial Q biosynthesis, and given the high sequence and structural homologies with QueF-L [30] it was not unreasonable to consider that QueF-L might utilize preQ_0_ directly (Figure 2, bottom). The fact that G^+^ was formed in bacterial tRNA when QueF-L was expressed in a *ΔqueF* strain [26] is consistent with both proposals. Indeed, the bacterial TGT can utilize preQ_0_ as a substrate [31], and preQ_0_ nucleoside is detected in *ΔqueF* mutants [26,27]. While biochemical analysis of the *canonical* arcTGT has demonstrated that it is not able to utilize G^+^-base [32], our 3D homology models of the catalytic domains of arcTGT from Crenarchaeota that lack ArcS [26] revealed differences from the canonical arcTGT in the active sites [33,34] that might allow accommodation of the formamidino group of G^+^ base were it available. Thus, both free preQ_0_ and preQ_0_-tRNA were considered viable candidates as the natural substrate for QueF-L (Figure 2).

#### 2.2.1. Thioimide Formation with preQ_0_ and preQ_0_-tRNA

**Figure 3:**
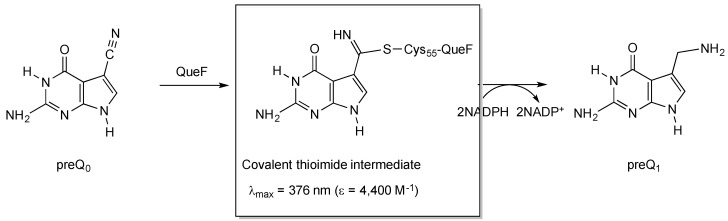
Structure and characteristics of the covalent thioimide intermediate formed in the QueF-catalyzed reaction in the biosynthesis of queuosine.

The conservation of Cys21 in QueF-L (*P. calidifontis* QueF-L numbering) and QueF (Cys55 in *Bacillus subtilis* QueF numbering) [26], which in QueF participates in the catalytic mechanism via nucleophilic attack of the thiol group on the nitrile of preQ_0_ to form a covalent thioimide intermediate (Figure 3) [28,35], suggested that QueF-L might utilize a similar intermediate in the mechanism to form the formamidine of G^+^. Given that the preQ_0_-QueF thioimide has a distinct absorption at 376 nm, we reasoned that probing for this absorption spectroscopically might allow us to determine whether preQ_0_ or preQ_0_-tRNA were capable of forming such an intermediate, and thus was the actual substrate.

**Figure 4:**
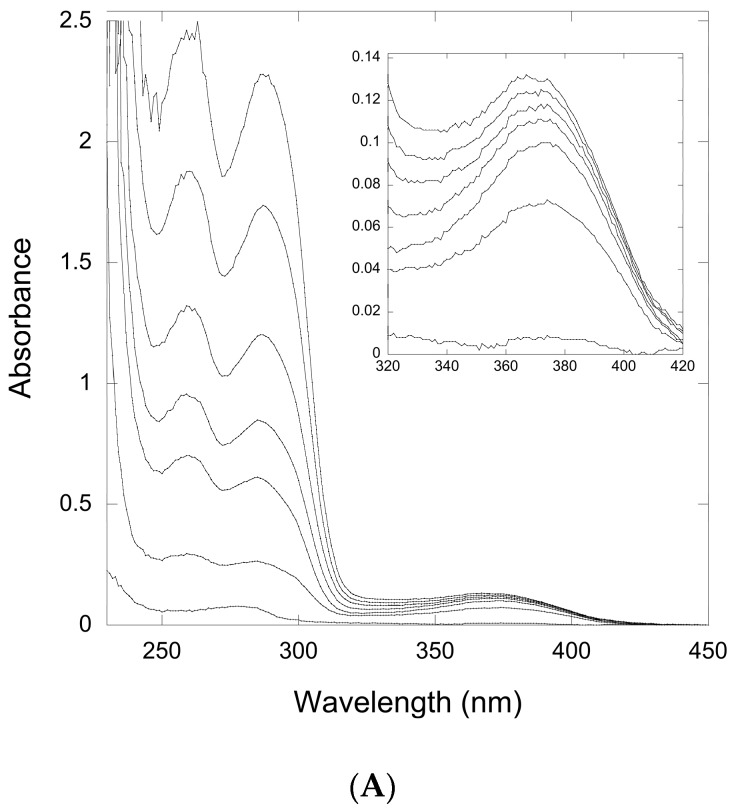

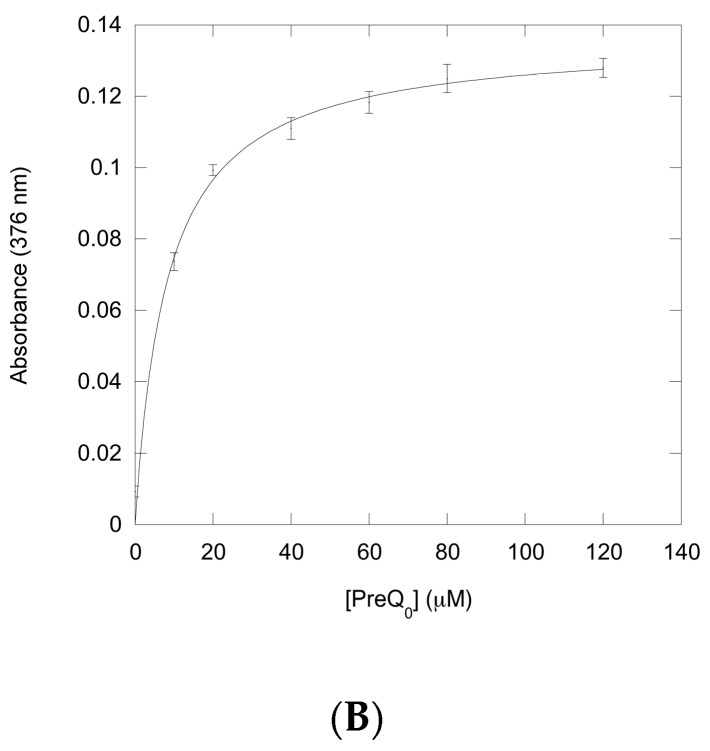
Ultraviolet-Visible (UV-Vis) spectroscopy of titration of a QueF-like with preQ0. (**A**) The thicker lower trace corresponds to the protein (20 μM) absorbance prior to preQ_0_ addition. The finer traces correspond, from bottom to top, to preQ0 concentrations of 10, 20, 40, 60, 80, and 120 μM, respectively. Inset shows the region around 376 nm expanded for clarity; (**B**) Plot of absorbance at 376 nm vs. [preQ_0_] exhibits a saturation curve.

**Figure 5:**
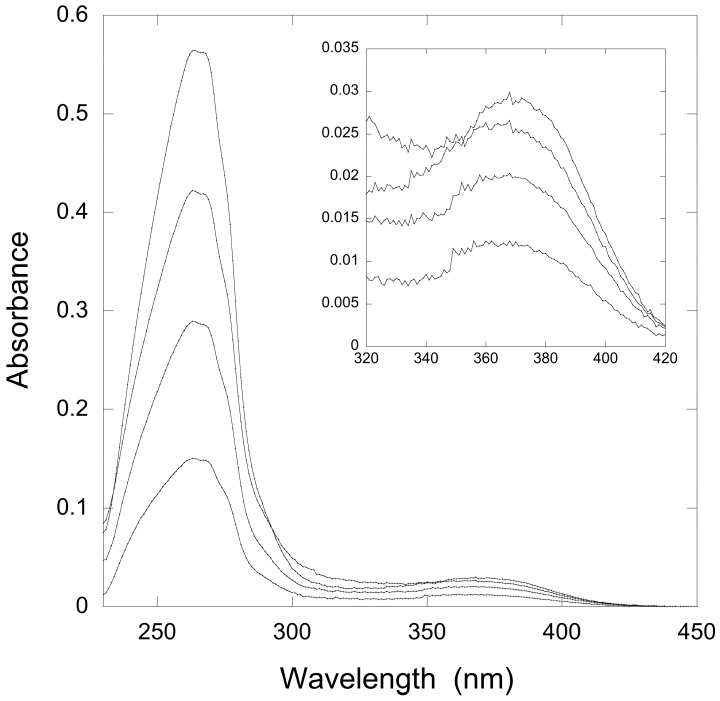
UV-Vis spectroscopy of a titration of QueF-L with preQ_0_-tRNA. The traces correspond, from bottom to top, to preQ_0_-tRNA concentrations of 1, 2, 3, and 4 μM, respectively. QueF-L was present at a concentration of 20 μM. Inset shows the region around 376 nm expanded for clarity.

Therefore, we titrated solutions of QueF-L with preQ_0_ or preQ_0_-tRNA and measured the absorption from 200 to 450 nm. Interestingly, titration with either potential substrate resulted in the formation of a new absorption at 376 nm that grew in intensity with increasing preQ_0_ or preQ_0_-tRNA (Figure 4A and Figure 5), suggesting that thioimide adducts formed with both. In the case of the preQ_0_ titration, the absorption exhibited a saturation curve (Figure 4B), consistent with specific binding to QueF-L (apparent *K*_D_ = 8 μM); due to limiting preQ_0_-tRNA we did not titrate this to saturation. Notably, these data are consistent with our subsequent observation of a thioimide intermediate in the X-ray structure of QueF-L crystalized in the presence of preQ_0_ [30].

#### 2.2.2. Reactivity of preQ_0_ and preQ_0_-tRNA Thioimides with Various Nitrogen Sources

The archaeosine base is very unstable, readily undergoing deamination to form preQ_0_ [32], making the direct observation of archaeosine base problematic if it was in fact the product of the reaction. However, the thioimide intermediate formed in the QueF-catalyzed reaction is quite stable [28,35], and this adduct can be isolated and probed free of excess substrate. Therefore, if the QueF-L adducts with preQ_0_ and preQ_0_-tRNA were similarly stable, it would be possible to investigate their fate when incubated in the presence of different ammonia sources.

To test this, we preformed the thioimide adducts of QueF-L with preQ_0_ and preQ_0_-tRNA and investigated their behavior with potential NH_3_ sources. Glutamine, and occasionally asparagine, function as NH_3_ donors in virtually all amidotransferases [36,37] with the concomitant formation of glutamate and aspartate, respectively, and therefore were potential sources of NH_3_ for QueF-L. However, all amidotransferases that utilize these amino acids as a source of NH_3_ also possess a conserved cysteine residue that is essential in the chemical mechanism for the overall hydrolysis of the amide groups, and QueF-L possesses only one conserved cysteine, which, as discussed above, is implicated in thioimide formation. Therefore, we also investigated NH_4_Cl as a source of free ammonia.

**Figure 6:**
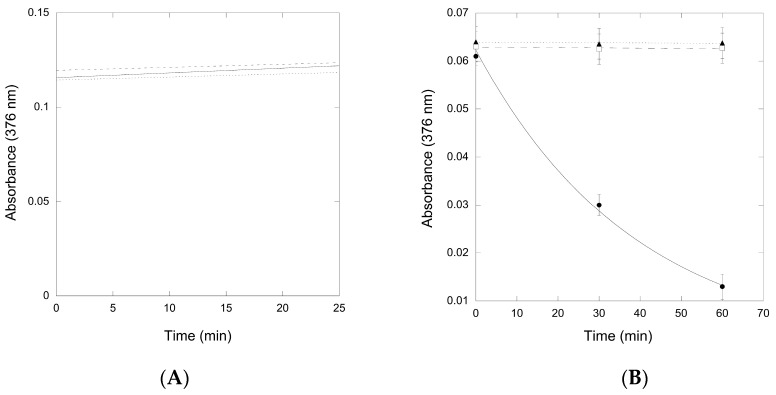
Time-course of the thioimide absorbance (376 nm) from QueF-L/preQ_0_ and QueF-L/preQ_0_-tRNA in the presence of various ammonia donors. (**A**) The absorbance of the QueF-L/preQ_0_ thioimide adduct in the presence of ammonium chloride (―), glutamine (---), or asparagine (^…^). Data show an example of a single experiment with each ammonia donor; (**B**) The absorbance of the QueF-L/preQ_0_-tRNA thioimide adduct in the presence of ammonium chloride (●,―), glutamine (□,---), or asparagine (▲,^…^). Data represents the average of three assays, each carried out in replicate with each potential ammonia donor (error bars represent the standard error (SE)).

The thioimide absorbance due to the QueF-L/preQ_0_ adduct was stable in the presence of all three potential sources of ammonia (Figure 6A), indicating that the thioimide was unreactive even when incubated for prolonged periods of time. Similarly, the absorbance due to the QueF-L/preQ_0_-tRNA adduct was stable in the presence of glutamine and asparagine, but in contrast it decayed rapidly in the presence of NH_4_Cl, consistent with turnover to form G^+^-modified tRNA (Figure 6B).

#### 2.2.3. Conversion of preQ_0_-Modified tRNA to G^+^-Modified tRNA by QueF-L

**Figure 7:**
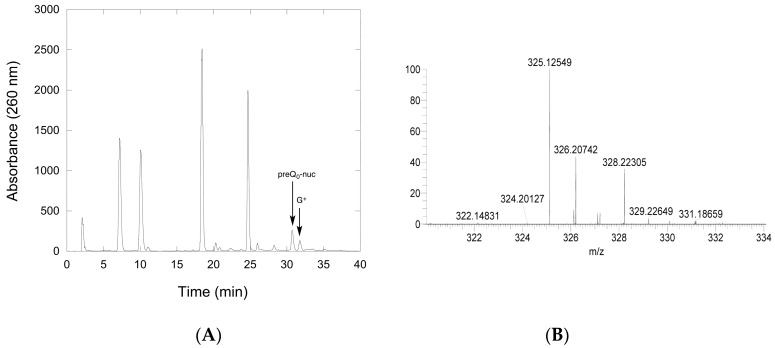
Confirmation of G^+^ synthesis by QueF-L. (**A**) An example of the HPLC chromatogram of nucleoside components of a tRNA^Gln^ transcript modified by incorporation of preQ_0_ with arcTGT, followed by reaction with QueF-L in the presence of NH_4_Cl. The tRNA was digested and dephosphorylated prior to HPLC analysis. (**B**) Partial mass spectrum of the component eluting at approximately 32 min and labeled G^+^ in the chromatogram. The peak at *m/z* 325.12549 is consistent with G^+^, and the peak at *m/z* 326.20742 with the isotopic M+1 ion.

To provide unambiguous evidence that preQ_0_-modified tRNA was the substrate of QueF-L, and that it was turned over in the presence of NH_4_Cl to produce G^+^-modified tRNA, we carried out reactions of QueF-L with preQ_0_-tRNA and NH_4_Cl, followed by isolation of the tRNA, and, after hydrolysis and dephosphorylation, analysis of the constituent nucleosides by liquid chromatography mass spectrometry (LCMS) A peak with the expected retention time of G^+^ was observed in the chromatogram (Figure 7A), and mass spectrometry analysis of that component provided an *m/z* of 325.12549 consistent with G^+^ (Figure 7B).

## 3. Discussion

The biosynthetic pathway to the 7-deazaguanosine modified nucleosides of tRNA is one of the most complex of the known modifications, and the only one in which a significant portion occurs outside the context of tRNA. While homologs of virtually all of the enzymes that catalyze steps in the pathway can be readily identified in all organisms that possess these modifications, the first and last steps of the pathways exhibit considerable diversity. Three distinct enzymes have been identified that catalyze the first step, although all are members of the same protein superfamily [22,24] and are thus evolutionarily related. In contrast, the enzymes catalyzing the last step in both the archaeosine [25,26] and queuosine [38,39] branches are structurally unique and represent distinct evolutionary solutions to the reactions that they catalyze. QueF-L is especially interesting in this regard as it is closely related to the bacterial QueF enzyme, an NADPH-dependent oxido-reductase in the queuosine pathway, and further expands the already diverse chemistry catalyzed by enzymes of the T-fold superfamily. 

Although in vivo data implicated *P. calidifontis* QueF-L as an amidinotransferase involved in the G^+^ pathway [26], there were two places in the pathway where it could conceivably function (Figure 2): in the last step analogous to ArcS [25], or earlier in the pathway prior to incorporation into the tRNA in analogy to QueF [27]. The results presented here clearly establish QueF-L as functionally analogous to ArcS, and preQ_0_-modified tRNA as the relevant substrate. Notably, in addition to the aforementioned new catalytic activity, this represents the first example of a T-fold enzyme utilizing a nucleic acid substrate.

**Figure 8:**
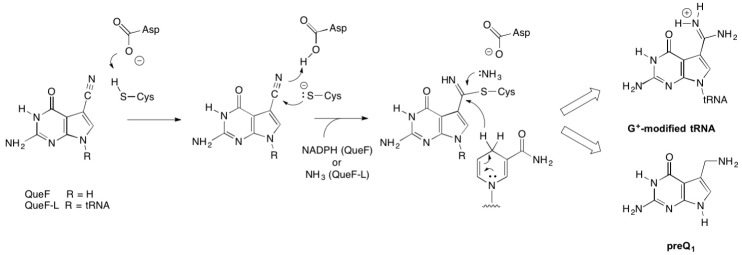
The common mechanistic steps leading to covalent thioimide intermediates in the reactions catalyzed by QueF and QueF-L.

While the products of the QueF- and QueF-L-catalyzed reactions are markedly different, an aminomethyl and a formamidine, respectively, both enzymes share an identical mechanistic path to analogous covalent thioimide intermediates (Figure 8), a process mediated by conserved active-site residues that include the cysteine involved in the thioimide and an aspartic acid that serves as a general acid/base [30]. The paths diverge at the thioimide intermediate, and differ in the identity of the nucleophile that attacks the thioimide intermediate—a hydride from NADPH in the case of QueF, and ammonia in the QueF-L reaction—and in the stoichiometry of co-substrate binding: two equivalents of NADPH to carry out the four-electron reduction, and one equivalent of ammonia.

Finally, unlike ArcS, as well as transamidases as a whole, glutamine is not the ammonia donor in the reaction, and instead the enzyme is only able to utilize free NH_4_^+^. The structural basis for this is now evident, as the X-ray crystal structure [30] shows that NH_4_^+^ gains access to the putative ammonia binding site via the central tunnel of the QueF-L decamer, which displays a uniform surface that clearly lacks any architectural features of an active-site that might bind a substrate that could serve as an ammonia donor. However, while the data presented here establish that QueF-L itself utilizes only NH_4_^+^, the data does not preclude the potential involvement of a protein partner that might function in generating NH_4_^+^ in vivo.

## 4. Materials and Methods

### 4.1. General

Buffers, salts and reagents (highest quality grade available), as well as gel filtration molecular weight standards and NTPs, were purchased from Sigma (St. Louis, MO, USA). Dithiothreitol (DTT), isopropyl-β-d-thiogalacto-pyranoside (IPTG), kanamycin sulfate, diethylpyrocarbonate (DEPC), and ampicillin were purchased from RPI Corporation (Chicago, IL, USA). [8-^14^C]-guanine was obtained from Perkin Elmer (Waltham, MA, USA). Amicon Ultra 15 and 0.5 centrifugal filter units and NovaBlue Singles competent cells were acquired from EMD Millipore (Billerica, MA, USA). Nickel-nitrilotriacetic acid agarose (Ni^2+^-NTA agarose), silica TLC plates, and Whatman GF-B PVDF syringe filters were purchased from Fisher Scientific (Pittsburgh, PA, USA). GeneJet Plasmid Miniprep kits, Klenow enzyme and PageRuler pre-stained protein ladder were purchased from Fermentas (Glen Burnie, MD, USA). Custom oligonucleotides were obtained from Integrated DNA Technologies (San Diego, CA, USA). Dialysis was carried out in Slide-A-Lyzer cassettes (ThermoFisher, Waltham, MA, USA). All reagents for sodium dodecyl sulfate polyacrylamide gel electrophoresis (SDS-PAGE) were purchased from BioRad (Hercules, CA, USA). SDS-PAGE analysis was carried out using 12% gels and visualized with Coomassie Brilliant Blue. Diethylpyrocarbonate-treated water was used in the preparation of all solutions for RNA-related assays. DNA sequencing was carried out at the DNA Services Core at Oregon Health and Sciences University (OHSU), Portland, OR, USA.

### 4.2. Enzymes

Bacterial alkaline phosphatase, nuclease P_1_ from *Penicillium citrinum,* snake venom phophodiesterase I and DNase were purchased as lyophilized powders from Sigma and stored in 50% glycerol with the appropriate buffer at the recommended temperature. *PfuUltra* DNA polymerase was obtained from Agilent (Santa Clara, CA, USA). Restriction enzymes were purchased from Fermentas (Glen Burnie, MD, USA) and New England Biolabs (Ipswich, MA, USA). Lysozyme was purchased from RPI Corporation (Mount Prospect, IL, USA). The plasmid encoding the Δ(172–173) variant of T7 RNA polymerase [40] was provided by John Perona. Recombinant *Methanocaldococcus jannashii* TGT was over-produced and purified as described previously [41].

### 4.3. Instrumentation

PCR was carried out on a 2720 Applied Biosystems cycler (Thermo, San Jose, CA, USA). UV-Vis spectroscopy was performed with a Cary 100 spectrophotometer (Agilent, Santa Clara, CA, USA) equipped with a thermostated cell holder. HPLC was carried out using an Agilent 1100 with photodiode array detector, and controlled via the Agilent Chemstation software, Agilent (Santa Clara, CA, USA). SDS-PAGE was carried out on a mini Protean III system from BioRad (Hercules, CA, USA). Mass spectrometry was performed on a LTQ-Orbitrap mass spectrometer (Thermo Electron, San Jose, CA, USA) equipped with an electrospray ionization (ESI) source in the Department of Chemistry’s core facility at Portland State University. Radioactivity was quantified with a Hidex 300 SL liquid scintillation counter (Turku, Finland) using Econo-Safe liquid scintillation cocktail (RPI).

### 4.4. PreQ_0_ Synthesis

PreQ_0_ was synthesized as previously described by Klepper (yield 12%, purity 98%). The product was purified by HPLC using a Luna C18 semi-preparative column (250 × 10 mm, 5 micron) from Phenomenex (Torrance, CA, USA) using a gradient of ammonium acetate (25 mM, pH 6.5) and acetonitrile (0–50% ACN over 30 min).

### 4.5. Cloning of *P. calidifontis queF-L*

The *queF-L* gene from *P. calidifontis* was synthesized (GenScript) with codon optimization for *E. coli* expression (see Supplemental Figure S1) and subcloned via PCR from into the FactorXa/LIC vector (Novagen) with the following primers:

Sense primer: 5′-GGTATTGAGGGTCGCATGCTGAAAGTCTCAAAAAGCC-3′
Antisense primer: 5′-AGAGGAGAGTTAGAGCCTTAGATGTAGACCGGCGGC-3′

PCR reactions contained 100 ng of linearized pGP358, 200 μM dNTPs, 50 pmol primers, 1× Pfu Ultra buffer (supplied by the manufacturer), and 2.5 units of Pfu Ultra DNA polymerase in a final volume of 50 μL. A three-step PCR thermocycling protocol was utilized: firstly, 94 °C for 3 min; secondly, 30 cycles of denaturation at 94 °C for 1 min, annealing at 50 °C for 1 min, and extension at 72 °C for 2 min; and thirdly, 72 °C for 3 min. The PCR products were then gel purified (1% agarose), and the DNA isolated (Qiagen PCR purification kit) and inserted into the FactorXa/LIC vector as described by the manufacturer. The primary structure of the resulting construct (pLBI14) was confirmed by capillary electrophoresis DNA sequencing at the OHSU DNA Services Core.

### 4.6. Over-Production and Purification of Recombinant *P. calidifontis* QueF-L

Luria-Bertani/kanamycin medium (3 mL) was inoculated with a single colony of *E. coli* BL21(DE3)/pLBI14 cells, and after 12 h of incubation at 37 °C a 1-mL aliquot was used to inoculate 100 mL of fresh LB/kan medium. The cultures were incubated at 37 °C and 250 rpm for 12 h and a 5-mL aliquot was taken and used to inoculate 500 mL of fresh LB/kan medium. When an optical density (OD)_600_ of 0.9 was reached, protein over-expression was induced by the addition of IPTG to a final concentration of 0.25 mM. The cell cultures were grown for an additional 4–5 h when the cells were collected by centrifugation at 7500 *g* for 15 min and frozen with liquid nitrogen. The cells were stored at −80 °C until further use.

The cells were resuspended to a density of 250 mg/mL in lysis buffer (50 mM Tris-HCl (pH 8.0), 300 mM KCl, 2 mM β-mercapto-ethanol (βME), and 1 mM phenylmethylsulfonyl fluoride (PMSF)). Lysozyme was added to a final concentration of 250 µg/mL and the cells incubated at 37 °C for 30 min, followed by three intervals of freeze-thaw cycles. DNase was added to a final concentration of 10 µg/mL and the cells were left at 37 °C for an additional 30 min. The cell lysate was centrifuged at 26,000 *g* for 30 min, and the cell-free extract (CFE) heated to 80 °C for 15 min followed by centrifugation at 26,000 *g* for 20 min. The resulting CFE was filtered using a low protein-binding 0.45-µm PVDF syringe filter, then loaded onto 5 mL of Ni^2+^-NTA agarose resin equilibrated in lysis buffer. The column was washed with five column volumes of lysis buffer followed by five column volumes of lysis buffer with 20 mM imidazole and no PMSF. The recombinant protein was eluted with seven column volumes of lysis buffer (with/out PMSF) containing 200 mM imidazole then concentrated to about 2 mL using the Amicon Ultra YM-10k and dialyzed overnight against 4 L of lysis buffer with no PMSF at 4 °C. Both proteins were cleaved by Factor Xa and purified as previously described. The cleaved protein was stored in 50% glycerol in 100 µL aliquots at −80 °C.

### 4.7. In Vitro Transcription of tRNA

Duplex DNA templates for in vitro transcription of *Methanobacterium thermoautotrophicum* tRNA^Gln^ were synthesized from two single-stranded oligodeoxynucleotides containing a complementary overlap region as previously described [42]. The oligonucleotide sequences used were:

5′-GCAGTAATACGACTCACTATAGGTCCCGTGGGGTAGTGGTAATCCTGCTGGGCTTTG-3′
5′-TGGTAGTCCCGAGCGGAGTCGAACCGCTGTCGCCGGGTCCAAAGCCCAGC-3′

The underlined region represents the T7 RNA polymerase promoter sequence. Transcription reactions were performed as previously described using the Δ(172–173) variant of T7 RNA polymerase [40], loaded onto a urea-PAGE gel, and after electrophoresis (80W, 60 min) the band was excised and extracted overnight in 100 mM ammonium acetate (pH 6.5) containing 1 mM EDTA. The gel was discarded and the tRNA precipitated from the remaining solution with three volumes of ethanol followed by cooling at −20 °C for 2 h. The solution was then centrifuged at 20,000 *g* for 20 min at 4 °C, the supernatant removed, and the RNA pellet washed with 70% cold ethanol. After centrifugation at 20,000 *g* the supernatant was removed and the tRNA was stored at −80 °C.

### 4.8. Preparation of preQ_0_ modified tRNA^Gln^

PreQ_0_ was inserted into the tRNA transcript using recombinant *Methanocaldococcus jannaschii* TGT. A solution of tRNA in succinate buffer (100 mM, pH 5.5) was refolded before use [43]. An aliquot of *Mj*TGT (10 µM) was added to a 1-mL solution containing 50 mM succinate (pH 5.5), 20 mM MgCl_2_, 100 mM KCl, 2 mM DTT, 100 µM tRNA, and 1 mM preQ_0_. After 45 min at 80 °C, the reaction was terminated by the addition of one-tenth volume of 2 M NaOAc (pH 4.0) followed by one volume of water-saturated phenol and one fifth volume chloroform:isoamyl alcohol (49:1). After vortexing for 20 s, the solution was centrifuged in a fixed angle rotor at 9000 *g* for 1 min. The aqueous phase was recovered and mixed with an equal volume of chloroform:isoamyl alcohol. After vortexing for 20 s, the solution was centrifuged in a fixed angle rotor for 1 min at 9000 *g*. The aqueous phase was recovered and concentrated using an Amicon Ultra4 centrifugal concentrator (EMD Millipore, Billerica, MA, USA). Subsequently, the preQ_0_-tRNA^Gln^ was precipitated from the retentate by the addition of three volumes of ethanol and cooling at −20 °C for 2 h. The solution was centrifuged at 20,000 *g* for 20 min at 4 °C, the supernatant removed, and the RNA pellet washed with 70% cold ethanol. After centrifugation again at 20,000 *g* the supernatant was removed and the preQ_0_-tRNA was resuspended in 3 mM sodium citrate (pH 6.3) and stored at −20 °C.

### 4.9. Guanine Incorporation Controls

To quantify preQ_0_ incorporation into tRNA^Gln^ a control reaction was run in which [8-^14^C]-guanine (50 mCi/mmol) was incorporated into the tRNA using *M. jannaschii* TGT and the tRNA isolated as described above. After quantifying the radiochemical specific activity of the [^14^C]tRNA, preQ_0_ was incorporated into the tRNA and aliquots of the reaction taken over time to measure the loss of [8-14C]-guanine (and incorporation of preQ_0_). The tRNA from each aliquot was precipitated and collected on Whatman GF/B glass filters. The filters were washed with cold ethanol in a vacuum filtration system so as to remove any unbound radioactive material. Once dry, the filters were placed in 7 mL scintillation vials with scintillation cocktail and the radioactivity was measured by scintillation counting.

### 4.10. Substrate Titration Studies

Titrations of QueF (20 µM) with preQ_0_ (3 mM in dimethylsulfoxide) or preQ_0_-tRNA were carried out in solutions containing 100 mM phosphate (pH 6.5), 50 mM KCl, 20mM MgCl_2_, and 1 mM DTT, while monitoring the absorbance from 230 to 450 nm. For the preQ_0_ titrations the concentrations of preQ_0_ ranged from 10 to 120 µM and the final concentration of DMSO did not exceed 4% of the total volume. For the preQ_0_-tRNA titrations the concentrations of preQ_0_-tRNA ranged from 1.0 to 4.0 µM.

### 4.11. Amidinotransferase Assays

Assays of amidinotransferase activity were carried out using QueF-L (20 µM) in 100 mM phosphate (pH 6.5), 50 mM KCl, 20 mM MgCl_2_, and 1 mM DTT, along with 1 mM preQ_0_ or 10 µM preQ_0_-tRNA, and incubation for 15 min at 37 °C. For the preQ_0_-tRNA assays NH_4_Cl (1 mM), glutamine (1 mM), or asparagine (1 mM) was then added while monitoring the absorption of the covalent thioimide adduct at 376 nm. For the assays with preQ_0,_ the solution was filtered through a centrifugal concentrator (Amicon) in a fixed angle rotor at 8500 rcf for 10 min and the retentate washed with buffer to remove excess preQ_0_. After 2× washes the retentate was reconstituted with buffer and NH_4_Cl/glutamine/asparagine was added while monitoring the loss of covalent thioimide adduct at 376 nm.

### 4.12. LCMS Analysis of QueF-L Assays with preQ_0_-tRNA

QueF-L (20 µM) was incubated in 100 mM phosphate (pH 6.5), 50 mM KCl, 20 mM MgCl_2_, 1 mM DTT, 20 µM preQ_0_-tRNA and 1 mM NH_4_Cl for 1 h at 37 °C. The tRNA was then precipitated using ethanol and digested using nuclease P1, snake venom phosphodiesterase and alkaline phosphatase and the nucleoside products of the reaction were analyzed by HPLC as previously described [44], using a Gemini C18 column (5 µm, 110 A, 2 × 250 mm; Phenomenex, Torrance CA, USA), with a mobile phase comprised of a linear gradient from 100% 25 mM NH_4_OAc (pH 6.3) to 85% 25 mM NH_4_OAc/15% acetonitrile developed over 30 min, then to 50% acetonitrile at 45 min. Flow was maintained at 0.3 mL/min. LCMS analysis of nucleosides was performed on an Orbitrap-LTQ mass spectrometer (Thermo Electron, San Jose, CA, USA) utilizing electrospray ionization (ESI). The ESI interface was operated in the positive mode using the following settings: end plate offset −500 V, capillary voltage −4500 V, nebulizer gas 1.6 bar, dry gas 4 L/min, dry temperature 200 °C, funnel 1 RF 350 Vpp, funnel 2 RF 350 Vpp, hexapole RF 400 Vpp, collision energy 10 eV and collision RF 300 Vpp.

## Supplementary Materials

The following are available online at http://www.mdpi.com/2218-273X/7/2/36/s1, Figure S1: Sequence of synthetic gene used for the production of *P. calidifontis queF-L*.
