# Supplementary material for: QueF-Like, a Non-Homologous Archaeosine Synthase from the Crenarchaeota
Source: Biomolecules. 2017 Apr 6;7(2):36. doi: 10.3390/biom7020036 (PMC5485725; doi:10.3390/biom7020036)
Supplement: Supplementary file 1 [file biomolecules-07-00036-s001.pdf]

ATGCTGAAAGTCTCAAAAAGCCCGAGCCTGGTCCGCCTGAAAACCCGTGGTGA
AAGCGTCTGCCCGATCTCAAAAACCGTCGATTCGTTTGAAGTGAGCGTTGAATA
TATTCCGCGTGGCGCAGTTCTGGCTATCGAAGAATTTAAGAAAATGGTCGATTCT
TACCGTGGTCGCGAAATTCTGCATGAAGAACTGGCCGTTGACCTGCTGGAAAAA
GTCAAAGCGGCCGTGAACCCGCCGTATGTCAAAGTGACCGTTAAAAGTTACTAC
ATTGGTGTTGAAGTGGAAGTCGTTGCCGAATCAGGTGGTGTGCCGCCGGTCTAC
ATCTAA

Figure S1. Sequence (5′→3′) of synthetic gene used for the production of *P. calidifontis queF-L.*
